# Multiple intracranial and spinal metastases from a nonfunctioning pituitary adenoma following multiple surgeries: an illustrative case with 16 years of follow-up

**DOI:** 10.1186/1477-7819-12-380

**Published:** 2014-12-12

**Authors:** Jun Wang, Er-meng Ma, Peng-fei Wu, Bo Qiu and, Yun-jie Wang

**Affiliations:** Department of Neurosurgery, The First Hospital of China Medical University, No. 155, Nanjing North Street, Heping Ward, Shenyang, 110001 China

**Keywords:** Metastasis, Microsurgery, Pituitary adenoma

## Abstract

Pituitary adenomas are the third most common primary intracranial tumor; however, those with postoperative metastases are very rare and are classically considered as pituitary carcinomas. The field of neurosurgery has struggled with diagnosing and treating these unusual lesions. In this report, we retrospectively analyze the clinical features, imaging findings, pathological characteristics and prognosis of one patient with non-hormone-secreting pituitary adenoma who had multiple intracranial and spinal metastases and underwent four surgeries in a 16-year follow-up period. In addition, on the basis of the existing literature, we explore the underlying mechanisms of, as well as the preventive and therapeutic strategies used to treat, pituitary carcinomas and postoperative metastasis of pituitary tumors.

## Background

Pituitary adenomas are very common and rank third in terms of incidence among all primary intracranial tumors. However, primary pituitary adenomas with intracranial or extracranial metastases are very rare and are commonly defined as malignant pituitary tumors or pituitary carcinomas [1–7]. The field of neurosurgery has struggled with the diagnosis and treatment of these unusual lesions. In general, the prognosis of this disease is not optimistic. Therefore, it demands an early diagnosis and effective therapies. In this report, we present the case of one patient with non-hormone-secreting pituitary adenoma who had multiple intracranial and spinal metastases and underwent four surgeries in a 16-year period. In addition, on the basis of a literature review, we discuss the diagnostic, preventive and therapeutic strategies for malignant pituitary adenoma and postoperative metastasis of pituitary tumors.

## Case presentation

### History

**Figure 1:**
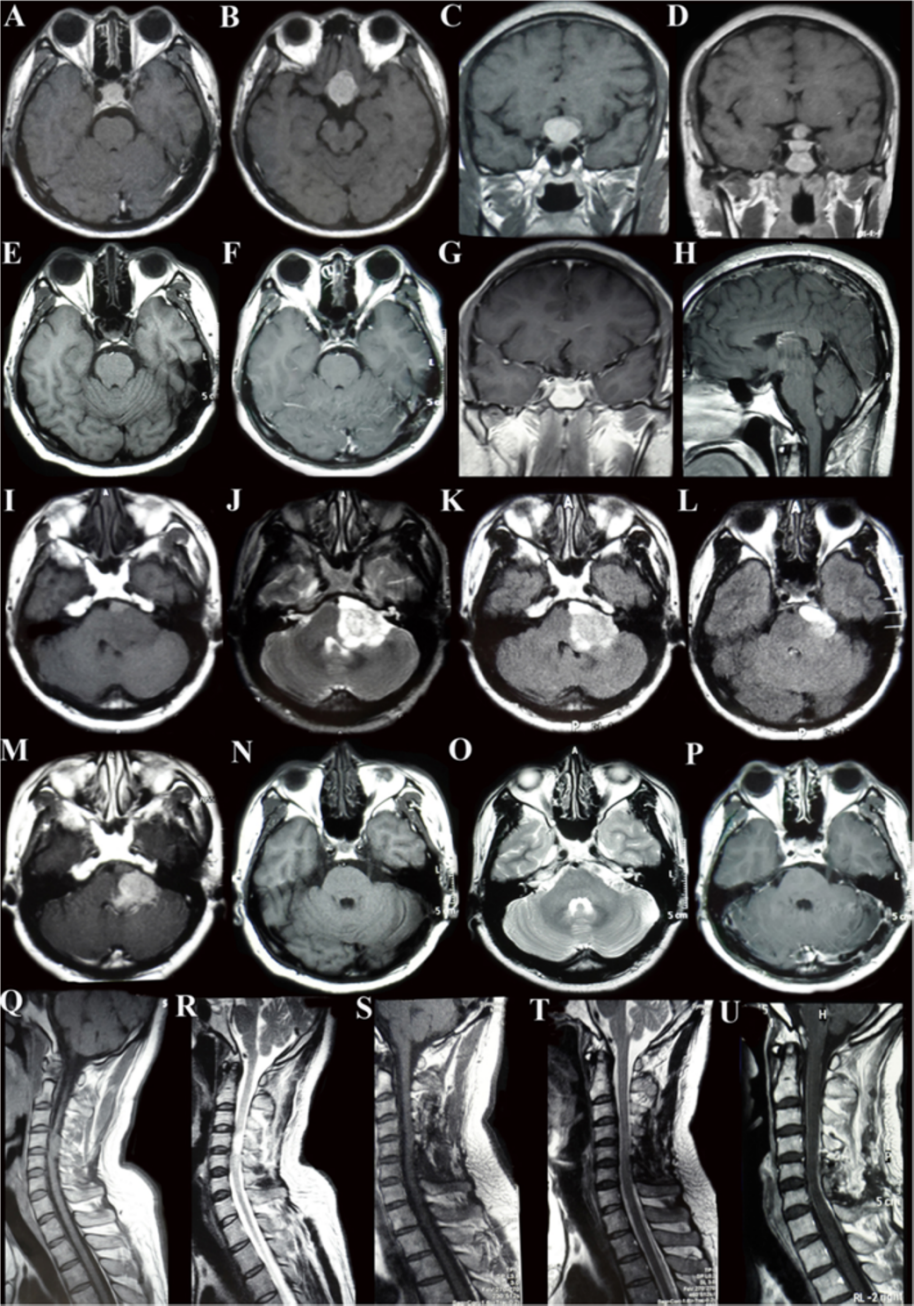
**Pre- and postoperative magnetic resonance imaging scans from the first three surgeries.** **(A to** **D)** Preoperative magnetic resonance imaging (MRI) scans obtained in December 1998 show a typical pituitary tumor. **(E to** **H)** and **(F to** **K)** Postoperative MRI scans show complete resection of the tumor (first surgery). **(I to** **M)** MRI scans taken in May 2008 show a lesion in the left cerebellopontine angle area. **(N to** **P)** Postoperative MRI scans show complete resection of the lesion (second surgery). **(Q and** **R)** MRI scans obtained in October 2010 show a lesion at the C4-C5 spinal level. **(S to** **U)** Postoperative MRI scans show complete resection of the lesion (third surgery).

A 40-year-old man was hospitalized (the fourth admission) with a major complaint of declining visual acuity during the preceding 6 months. He underwent his first craniotomy in 1998 for removal of a large pituitary adenoma (Figure 1). The final pathological diagnosis was benign pituitary adenoma. In 2008 and 2010, he was readmitted to our hospital with hearing loss in the left ear and numbness and weakness in his limbs, and the second and third surgeries were performed for the removal of lesions at different sites, respectively (Figure 1). Generally, the histological examinations of all surgical specimens obtained from the second and third operations showed typical benign pituitary adenomas. After the third operation, whole-brain and spinal cord radiation therapy was recommended because the tumor was then clinically diagnosed as a malignant lesion due to its behavior of recurring metastases. Unfortunately, because of the possible side effects, the patient refused to undergo the recommended treatment. On physical examination at the fourth admission, the patient was conscious and could answer questions correctly. Mild bilateral papilledema was detected. The patient had bitemporal hemianopsia and declining bilateral visual acuity (left: 4.8, right: 5.0). He had no other neurological deficit.

### Laboratory and neuroimaging examinations

**Figure 2:**
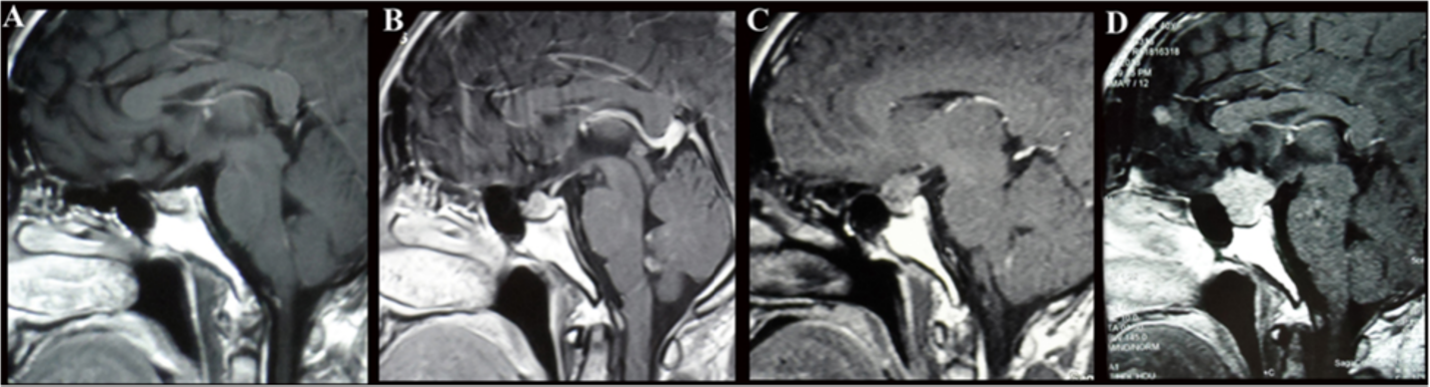
**Magnetic resonance imaging scans showing the recurrence of the pituitary tumor. (A)** Postoperative magnetic resonance imaging (MRI) scan taken in September 2008 (10 years after the first surgery) show no evidence of recurrence. **(B)** Postoperative MRI scan obtained in June 2009 show the recurrence of the tumor. **(C and** **D)** Postoperative MRI scans obtained in December 2011 and May 2013, respectively, show rapid growth of the tumor.

**Figure 3:**
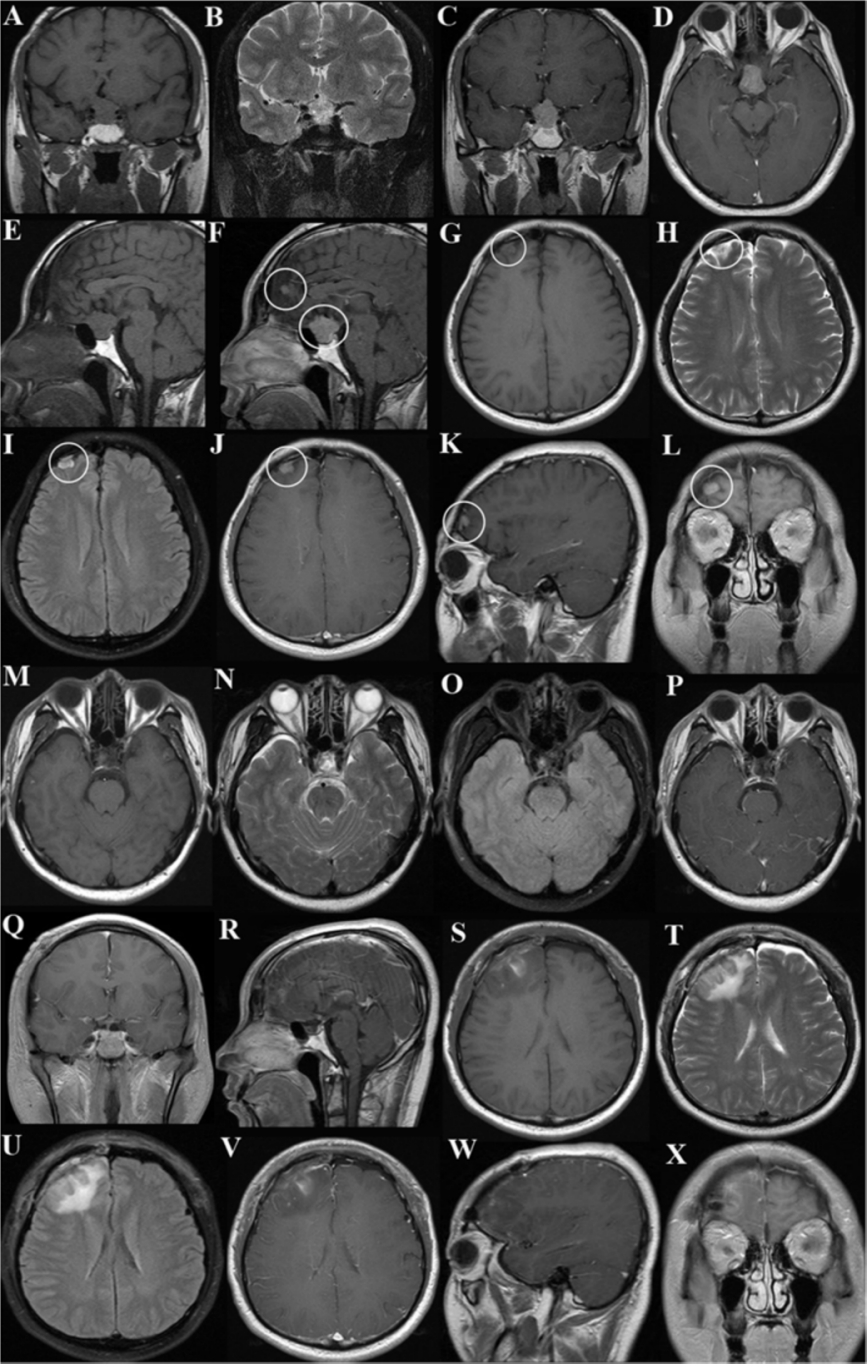
**Pre- and postoperative magnetic resonance imaging scans from the fourth surgery.** **(A to** **L)** Preoperative magnetic resonance imaging (MRI) scans show three intracranial lesions (circles). **(M to** **X)** Postoperative MRI scans show complete resection of all lesions.

The laboratory findings in May 2013 (at the time of the patient’s fourth admission) showed that his serum hormone levels, including prolactin (PRL) (225.0 mIU/L; reference ranges, 53.0 to 360.0 mIU/L), adrenocorticotropic hormone (ACTH) (47.43 pg/ml; reference range, 7.20 to 63.30 pg/ml), human growth hormone (hGH) (0.22 mIU/L; reference range, 0.16 to 2.60 mIU/L), insulin-like growth factor 1 (1.01 U/ml; reference range, 0.5 to 2.0 U/ml) and thyroid-stimulating hormone (TSH) (2.243 mIU/L; reference range, 0.350 to 4.940 mIU/L), were all normal. His visual field examination indicated bitemporal hemianopsia. Contrast-enhanced magnetic resonance imaging (MRI) of the patient’s brain indicated the presence of three intracranial lesions, with their locations being in the sella turcica (maximum diameter, 2.0 × 1.2 × 2.2 cm), the right frontal lobe (maximum diameter, 0.5 × 0.4 × 0.3 cm) and the anterior cerebral falx (maximum diameter, 0.6 × 0.5 × 0.3 cm), respectively. All lesions demonstrated long T1 and T2 heterogeneous signals and high signal intensity on fluid-attenuated inversion recovery images with significant enhancement (Figures 2 and 3).

### Surgical procedures

In the patient’s fourth surgery, the right subfrontal approach of craniotomy was adopted, and the incision was made at the same site used in the first surgery in 1998. After we expanded the bone window to the middle line and attempted to cut the dura mater open, we observed the dura mater to be tightly adhered to the right frontal cortex, which was then carefully dissociated under a microscope. During the dissociation process, we first exposed and removed the lesion in the right frontal lobe. The lesion was gray and soft, with a base connected to the dura mater. The lesion was immediately sent for intraoperative pathological examination, and the diagnosis was neuroendocrine neoplasm. Subsequently, we dissociated and removed the tumor in the sellar region. We then exposed and resected the tumor at the cerebral falx using an interhemispheric fissure approach. After the surgery, the patient had a satisfactory recovery of visual field, but experienced transient diabetes insipidus and hypopituitarism, from which he gradually recovered after symptomatic treatment. No other operation-related complications occurred. The postoperative MRI demonstrated complete removal of three lesions. Whole-brain and spine radiotherapy in combination with chemotherapy were suggested to the patient, but he rejected this suggestion.

### Postoperative pathology

**Figure 4:**
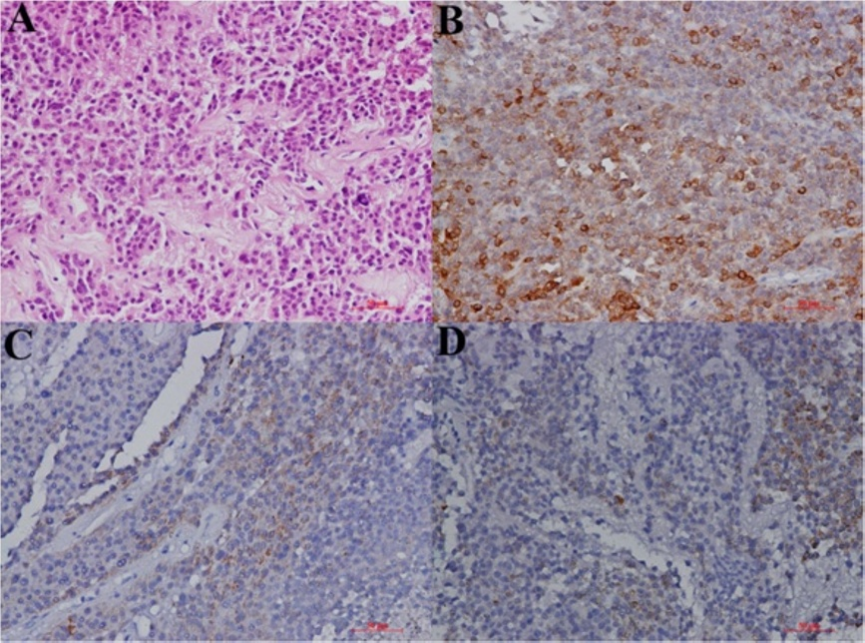
**Postoperative pathological studies. (A)** Hematoxylin and eosin–stained sections (original magnification, ×200) show large nuclei and clear heteromorphism. **(B to** **D)** Immunohistochemical specimens show positive staining for adrenocorticotropic hormone (low +) **(B)** and negative staining for growth hormone **(C)** and prolactin **(D)**.

Pathological examinations indicated that the three lesions were all pituitary adenomas with atypical cellular proliferation. The results of the immunohistochemical examinations were ACTH (+), hGH (low +), PRL (low +) and Ki67 (+; <2%) (Figure 4).

### Follow-up

The patient was followed from December 1998 (after the first surgery) to December 2014. He achieved a satisfactory recovery after each surgery. No tumor recurrences or metastatic lesions were observed in the sellar region or other regions during the 19-month follow-up period from May 2013 (after the fourth surgery) to December 2014.

## Discussion

Pituitary tumors are very common, and most are benign. The World Health Organization (WHO) classification of tumors of the central nervous system (2000) categorizes pituitary tumors into three grades: benign, intermediate or atypical, and malignant [7]. It is worth noting that the pathological classification assigned should be closely associated with clinical biological behaviors. Therefore, the WHO standards state that, even if pathological analyses reveal that the primary or metastatic lesion of pituitary tumor that has metastasized to subarachnoid space or extracranial sites is benign, it still belongs to the malignant pituitary tumor classification for its aggressive biological behavior [7–12]. Pituitary carcinoma, the most common form of malignant pituitary tumors, is also defined by the presence of a pituitary tumor that either is not contiguous with the primary sellar tumor and/or is not a pituitary tumor that has metastasized to sites distant from the pituitary [2, 4, 13]. This terminology is commonly used for describing the malignant pituitary adenoma. Currently, there is still controversy regarding the initiation and progression of pituitary carcinoma, the focus of which is whether it develops from benign adenomas or occurs *de novo* and whether the hormonal subtype does affect tumor aggressiveness, treatment outcome and prognosis [1]. Although the progression from benign pituitary adenomas to carcinomas has often been considered [1, 6, 14, 15], the detailed mechanisms underlying this phenomenon are still unclear and may involve multiple molecular events.

A literature review revealed that pituitary carcinoma is very uncommon and accounts for only approximately 0.1% to 0.2% of all pituitary tumors [2, 3, 15–17]. According to a recent study, most of the reported pituitary carcinomas are functional (83%), with 35% of the lesions producing ACTH, 33% PRL, 9% growth hormone (GH), 4% luteinizing hormone (LH) and/or follicle-stimulating hormone (FSH), and only 1% TSH [1]. Nonfunctioning tumors represent 19% of all cases, also including silent ACTH, FSH, LH and rare null-cell pituitary carcinomas [1]. Similar results were reported in another study, with the majority (36%) of the hormonally active tumors producing PRL; 30% secreting ACTH; 5% producing GH; 2% producing TSH, gonadotropin-releasing hormone or LH; and 23% not secreting any hormones [18]. The endocrine parameters (hormone in blood serum) in the case of our patient were clinically normal. However, the immunopathological examination revealed positive ACTH staining. Hence, the possibility of an ACTH adenoma that did not affect endocrine function could not be ruled out clinically. As reported previously, corticotrophic carcinomas develop in the setting of “silent” corticotrophic tumors in approximately 25% cases [2]. Similarly, it remains unclear whether a metastatic functional pituitary adenoma can possess endocrine function.

The spread pathway of tumor cells from a pituitary carcinoma has not been determined, but has been proposed to be varied, including cerebrospinal fluid circulation and lymphatic or hematogenous metastasis to extracranial sites [19, 20]. The metastasis of pituitary carcinomas might be related to the biological properties of tumors, such as invasion, as well as to the surgical procedures used. According to the existing literature, the metastases of pituitary tumors mostly occur after surgery, in particular after craniotomy. As reported by Tanaka *et al*., the development of metastases followed surgery performed for primary pituitary tumors in 21 (70%) of 30 cases [21]. Therefore, it was suggested that surgery may precipitate postoperative metastasis of pituitary adenomas. From a neurosurgeon’s point of view, this may be due to several reasons. First, neurosurgery, especially a craniotomy, not only can rupture the tumor capsule but also can injure the normal arachnoid membrane, creating favorable conditions for the spread of tumor cells along the subarachnoid space. Second, the vessels of or around the tumor are destroyed, which may enhance hematogenous metastasis. Third, tumor cells may be disseminated by the surgical instruments or flush water. Moreover, we noticed that most patients with postoperative metastases of pituitary tumors reported in the literature underwent craniotomies before 2000 [5, 9–12, 22–27]. After 2000, because of the progress made with the use of the endonasal transsphenoidal surgical technique, this approach has been applied in more than 90% of pituitary tumor surgeries. The endonasal transsphenoidal approach rarely causes a massive cerebrospinal fluid leakage or tumor cell dissemination to the subarachnoid space, and, as a result, the possibility of surgery-associated postoperative tumor metastasis in the subarachnoid space is significantly reduced. On the basis of this analysis, it is indicated that postoperative metastasis of pituitary tumors may be closely related to the surgical methods employed. However, the discussion above lacks the support of randomized controlled studies with large sample sizes. Moreover, the case sample with postoperative pituitary tumor metastasis is relatively low. Therefore, the analyses above are clinical inferences that require further verification.

The treatment for pituitary carcinoma should be comprehensive, including neurosurgery, radiotherapy, chemotherapy and medical therapy [1–3, 15–17]. Neurosurgery has been considered to be the first line treatment for pituitary carcinoma, for it not only can relieve the clinical symptoms by removing the lesions but also can aid in the diagnosis by providing pathological samples [1, 2, 15]. Although it is considered that surgery alone is rarely curative, especially for patients with multiple intracranial or systemic metastases, it should be pointed out that surgery can notably prolong survival in some cases [28]. Our patient is a case in point, because he underwent four surgeries in the 16-year follow-up period and had a satisfactory recovery after each surgery. Individualized surgical plans should be developed for the treatment of pituitary carcinoma, including transsphenoidal surgery, transcranial surgery and combined surgeries. Transsphenoidal surgery may be preferable in most cases, except for those with multiple intracranial metastatic lesions or large pituitary carcinomas with invasiveness into parasellar structures, for whom transcranial surgery should be recommended.

In our patient, three intracranial lesions were found in the MRI examination. Because all the lesions were located supratentorially, a one-stage transcranial operation was performed to remove all three lesions simultaneously. Furthermore, although the lesions were intracranial, they were all located at sites outside the brain; therefore, it was feasible to remove the lesions with minimal invasion of the brain tissue. The outcome of the surgery was satisfactory, with no serious postoperative complications. After surgery, radiation therapy, such as fractionated radiation therapy, stereotactic radiosurgery and whole-brain or spinal radiation, should be administered in most cases to prevent tumor relapse [1, 2, 15, 29]. In patients with tumors subtotally resected or with multiple intracranial metastases, radiation therapy should be performed earlier to slow growth of the tumor and/or metastatic deposits. Clinically, radiation therapy has been suggested to be effective in achieving local control or slowing tumor growth in some cases [1, 15, 29]. However, because these results lack evidence derived from randomized controlled trials, the usefulness of radiation therapy should be determined in further studies.

Chemotherapy is commonly used in the treatment of many types of carcinomas; however, its application in the treatment of pituitary carcinoma is just now being tested. The most commonly used drugs are temozolomide and 1-(2-chloroethyl)-3-cyclohexyl-1-nitrosourea in combination with 5-fluorouracil [2, 30]. Other agents used include cisplatin, etoposide, cyclophosphamide, adriamycin, methotrexate, vinblastine and bleomycin. Although various chemotherapy protocols have been applied in the treatment of pituitary carcinomas, no significant effect on tumor size or secretion and only transient improvement or stabilization have been observed in a minority of patients [1, 15]. More important, because of the rarity of these lesions, the potential use of chemotherapeutic drugs in the treatment of pituitary carcinoma has not been verified in multicenter randomized controlled trials. Therefore, similarly to radiotherapy, the usefulness of chemotherapy also needs to be validated by future studies. Medical therapy, such as with bromocriptine and cabergoline, has been considered as the first-line treatment for functional pituitary adenomas (benign). In pituitary carcinomas, medical therapy has been used to control excess hormone secretion and/or tumor proliferation. Currently, the most widely used clinical drugs include dopamine agonists, octreotide and lanreotide, which have been used to treat PRL-, GH-, ACTH- and TSH-secreting pituitary carcinomas [1, 2, 15]. However, the clinical benefit was reported to be minimal. In general, the efficacy of medical therapy for pituitary carcinomas still needs to be determined. Future improvements in targeted molecular therapies (biotherapy or gene therapies) and immunotherapy may provide more effective options for treatment of malignant pituitary tumors.

## Conclusions

Pituitary carcinoma is very rare. The recommended treatment regimens are comprehensive, including neurosurgery, radiotherapy, chemotherapy and medical therapy. Scientific and professional follow-up is conducive to the early detection of tumor recurrence or postoperative pituitary tumor metastasis. To date, the effectiveness of treatments has not been satisfactory. Future progress in targeted therapy and immunotherapy may provide more effective treatments for this rare malignant lesion.

## Consent

Written informed consent was obtained from the patient for publication of this case report and any accompanying images. A copy of the written consent is available for review by the Editor-in-Chief of this journal.
